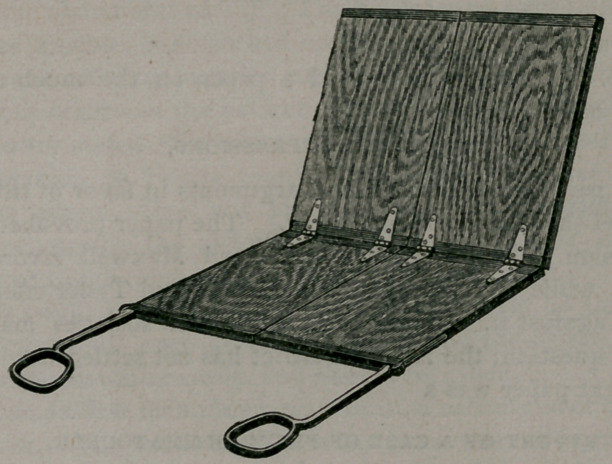# A Portable Gynecological Table

**Published:** 1889-10

**Authors:** L. H. Luce

**Affiliations:** Member Mass. State Soc., Formerly physician and consulting physician to St. Luke’s Hospital


For Daniel's Texas Medical Journal.

### A PORTABLE GYNECOLOGICAL TABLE.

BY L. H. LUCE, M. D., MEMBER MASS. STATE SOC.,
Formerly physician and consulting physician to St. Luke's Hospital.

THE importance of a suitable table for gynecological examinations is universally recognized by the profession. Many admirable stationary tables have been in use for sometime, such as the Wadsworth, Chadwick, Bennett and the University. These tables, however, are only available in the office of the physician, being too large of course for use at the bedside.

Having in twenty years' practice seen the necessity of a table that could be used equally well in the office of the physician or at the bedside, I have invented a portable table which can be carried in the hand or buggy as easily as a satchel.

From quite an extensive use now, it has proved so thoroughly useful even beyond my most sanguine expectations, that I desire to bring it to the notice of my professional brethren.

It is composed of four sections securely united by hinges, so as to allow of folding from before backward. The two upper sections are united by two hinges so as to permit folding laterally.

To the two lower sections are fastened the foot rests or stirrups, made of metal eight inches in length and provided with prop-joints to admit of adjustment at any angle, and also to fold back when not in use When open for use it is of the same dimensions as the stationary tables, being five feet long, including the foot rests, and two feet wide. Figure 1 gives a good illustration of it in this position.

When folded, it is twenty-two inches long, twelve inches wide and about three inches thick. It is well shown in this position by figure 2, while figure 3 shows it with the back raised at an acute angle. It is strongly made, compact and handsome, being made of the best material finely finished.

In placing pessaries, making applications to the cervix, in curetting the womb or for purposes of diagnosis, especially when used at the home of the patient, it is simply invaluable, and not inferior to the high-priced stationary tables.

When used at the office, it is unfolded and placed across the office table, when it becomes, for all practical purposes, a stationary table. A footstool, or small box, may be placed at the foot of the table, to aid the patient in mounting, which is done in precisely the came manner as when a stationary table is used.

When used at the home of the patient, as is so often necessary, especially in country practice, it may be placed across the bed, dining-room table, stand, or even on chairs. If used on the bed, it is best to place the table on a firm mattress, stretched across the bed, and cover it with a blanket. This insures the necessary firmness and comfort.

In these days, when every practitioner has to be a gynecologist, such a table is as much a necessity for a thorough examination and treatment of the female pelvic organs, as a stethoscope is in diseases of the chest.

It is also useful in examinations and operations on the rectum and genito-urinary organs.

It is simple, efficient, portable, and so cheap as to be within the reach of every one. This table is admirably made, by the well-known firm of Leach & Greene, Boston, Mass.

## Figures and Tables

**Figure f1:**
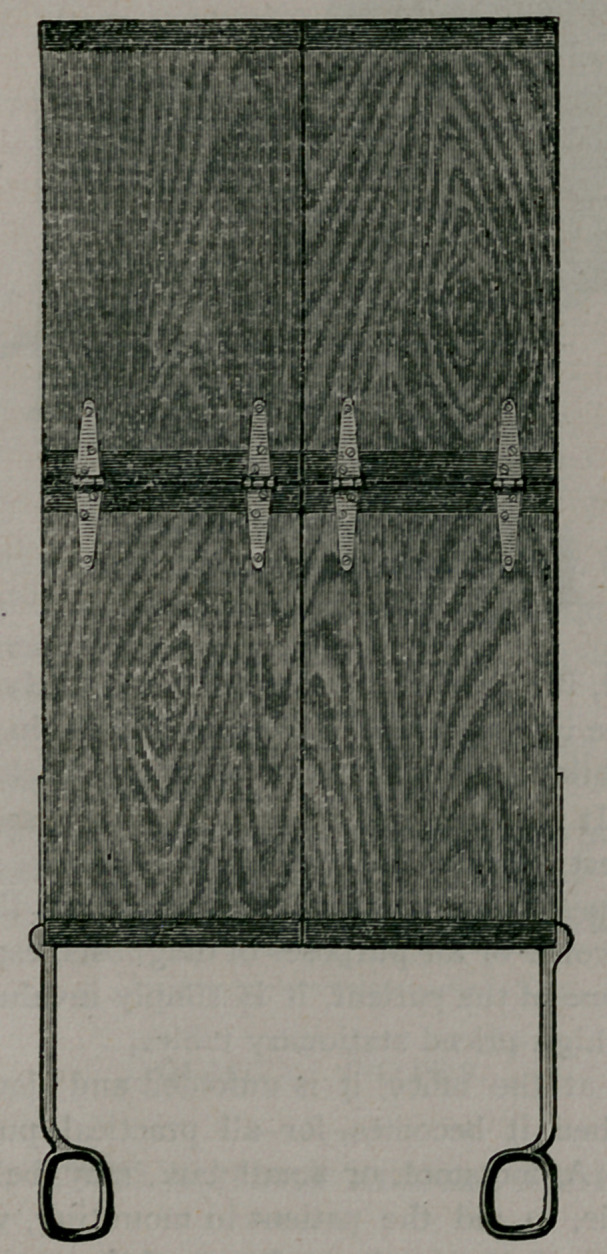


**Figure f2:**
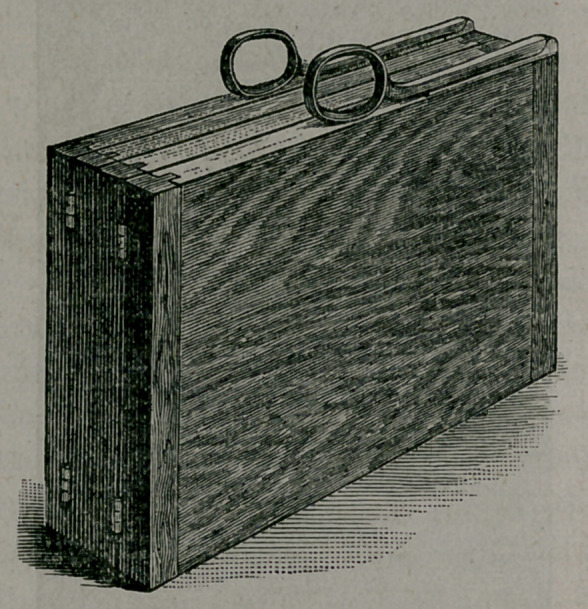


**Figure f3:**